# *Cymbopogon proximus* and *Petroselinum crispum* seed ethanolic extract/Gum Arabic nanogel emulsion: Preventing ethylene glycol and ammonium chloride-induced urolithiasis in rats

**DOI:** 10.1007/s00240-024-01559-2

**Published:** 2024-04-02

**Authors:** Hend A. Essa, Alaa M. Ali, Mona A. Saied

**Affiliations:** 1 Nutrition and Food Sciences Department, Food Industries and Nutrition Research Institute, National Research Centre, Dokki, Cairo, 12622 Egypt; 2 Department of Pathology, Faculty of Veterinary Medicine, Cairo University, Giza, 12211 Egypt; 3 Microwave Physics and Dielectrics Department, National Research Centre, Dokki, Cairo, Egypt

**Keywords:** Urolithiasis, *Cymbopogon proximus*, Parsley seed, Gum Arabic, Ethylene glycol, Renal dysfunction

## Abstract

Urolithiasis is a prevalent urological disorder that contributes significantly to global morbidity. This study aimed to assess the anti-urolithic effects of *Cymbopogon proximus* (Halfa Bar) and *Petroselinum crispum* (parsley) seed ethanolic extract /Gum Arabic (GA) emulsion, and its nanogel form against ethylene glycol (EG) and ammonium chloride (AC)-induced experimental urolithiasis in rats. Rats were divided into four groups: group 1 served as the normal control, group 2 received EG with AC in drinking water for 14 days to induce urolithiasis, groups 3 and 4 were orally administered emulsion (600 mg/kg/day) and nanogel emulsion (600 mg/kg/day) for 7 days, followed by co-administration with EG and AC in drinking water for 14 days. Urolithiatic rats exhibited a significant decrease in urinary excreted magnesium, and non-enzymic antioxidant glutathione and catalase activity. Moreover, they showed an increase in oxalate crystal numbers and various urolithiasis promoters, including excreted calcium, oxalate, phosphate, and uric acid. Renal function parameters and lipid peroxidation were intensified. Treatment with either emulsion or nanogel emulsion significantly elevated urolithiasis inhibitors, excreted magnesium, glutathione levels, and catalase activities. Reduced oxalate crystal numbers, urolithiasis promoters’ excretion, renal function parameters, and lipid peroxidation while improving histopathological changes. Moreover, it decreased renal crystal deposition score and the expression of Tumer necrosis factor-α (TNF-α) and cleaved caspase-3. Notably, nanogel emulsion showed superior effects compared to the emulsion. *Cymbopogon proximus* (C. *proximus*) and *Petroselinum crispum* (P. *crispum*) seed ethanolic extracts/GA nanogel emulsion demonstrated protective effects against ethylene glycol induced renal stones by mitigating kidney dysfunction, oxalate crystal formation, and histological alterations.

**Supplementary Information:**

The online version contains supplementary material available at 10.1007/s00240-024-01559-2.

## Introduction

Urolithiasis, commonly referred to as kidney or renal stone disease, is characterized by the accumulation of crystals within the renal tract. In recent times, it has emerged as the third most widespread issue affecting the urinary system, exerting a significant impact on public health over the past two decades [1]. While kidney stones are often perceived as non-life-threatening, they can have lethal consequences. These stones progressively damage the kidneys and can ultimately lead to chronic kidney disease [2]. Furthermore, the financial load associated with stone disease is considerable, with the expenses related to stone treatment and management rivaling those of combined treatments for prostate and bladder cancers [3].

Urinary stone disease has an ancient history in humans, with documented cases dating back to the Egyptian era. The historical record of urolithiasis is extensive, with evidence found in Egyptian mummies and references in ancient texts such as the Asutu in Mesopotamia, the Sushruta Samhita, and the Aphorisms of Hippocrates [4]. In contemporary times, urolithiasis remains a formidable challenge within the human population, with prevalence rates varying across different regions. Notably, it affects about 1–19% of individuals in Asia, 7–13% in North America, 5–10% in Europe, 4% in South America, and a striking 20–25% in Middle Eastern countries [5].

The epidemiology of urolithiasis is influenced by various factors, including geography, climate, ethnicity, diet, genetics, and demographic characteristics such as age, sex, and race [6]. The increased prevalence of kidney stones in developed countries is associated with elevated consumption of salt and protein, as well as the prevalence of metabolic syndrome. In contrast, in developing countries, it can be linked to malnutrition and inadequate water intake [5]. Therefore, there is a pressing need for novel medical prophylaxis strategies to address stone formation in the face of these diverse contributing factors.

To date, urolithiasis remains a challenging condition to treat effectively, owing to its intricate pathophysiology and multifaceted etiology. Across different ethnic groups worldwide, the historical use of various medicinal plants has garnered attention for their potential in managing urolithiasis. Many of these botanical resources have demonstrated promising effects. Significantly, medicinal plants often present an economically viable and regarded as a safe alternative for intervention. Recent investigations have assessed the antiurolithiatic efficacy of numerous plant species, as documented in studies [7–9].

*C. proximus*, a member of the Poaceae family, is a traditional medicinal plant in Egypt known as “Halfa Bar.” It has gained recognition within Egyptian traditional medicine for its dual functionality as both a diuretic and a renal antispasmodic [10]. Particularly in the southern regions of Egypt, this plant plays a significant role in traditional medicine, where it is utilized as a diuretic to enhance urine flow and as a remedy to aid in the expulsion of small urinary tract stones [11]. *C. proximus* serves as a valuable source of bioactive metabolites, encompassing terpenoids, and phenols. These compounds have found applications in pharmaceutical drug production and the cosmetics industry, as reported by Malin et al. in 2018 [12]. Moreover, recent studies conducted by Yagi et al. in 2020 [13] and Moglad et al. in 2020 [14] have shed light on its efficacy in combating human breast cancer and human colon adenocarcinoma cell lines, revealing promising anticancer properties in addition to its established antimicrobial activity.

*P. crispum*, known as Parsley, belongs to the Apiaceae family and is commonly referred to as “Baqdunis.” It boasts a rich history of medicinal utilization across European, Mediterranean, and Asian regions [15]. One of its distinctive features is the presence of small, dark seeds that contain a notably higher concentration of volatile oil when compared to its stems or leaves [16]. Parsley has garnered global recognition owing to its remarkable medicinal properties, primarily attributed to its potent antioxidant activity. This antioxidative potential can be ascribed to the presence of bioactive compounds like tocopherol, flavonoids, and carotenoids [17]. The therapeutic potential of parsley and its derivatives extends to a range of kidney-related ailments, making it a valuable candidate for complementary or alternative treatments [18]. Research has revealed its anti-inflammatory properties, edema mitigation capabilities, anti-hypertensive effects, anti-diabetic properties, and antimicrobial activity. Additionally, parsley enhances antioxidant defenses, modulates enzyme activities, elevates glutathione levels in the kidney, and contributes to the restoration of kidney tissue following nephrotoxicity [19].

GA is a viscous exudate obtained from the umbrella-shaped branches of *Acacia seyal* and *Acacia senegal*. This substance is extracted either by making an incision on the branches to allow the exudate to flow or by harvesting naturally occurring exudate, which subsequently hardens upon exposure to air. GA is predominantly sourced from regions such as Sudan, Chad, and Nigeria [20]. Historically, GA has been employed to address a range of medical conditions, including chronic renal disease and stomach pain. Recent investigations have illuminated various pharmacological and medical attributes associated with GA. These encompass anti-inflammatory properties and its capacity to safeguard nephrons [21]. Notably, GA stands out for its exceptional antioxidant activity. Moreover, research indicates that high doses of GA can enhance renal function, modulate specific serum minerals, bolster antioxidant enzyme activity, and prevent renal damage in rat models afflicted with diabetic kidney failure, all while maintaining safety [22].

Therefore, the quest for antilithiatic drugs derived from natural sources, known for their efficacy and minimal side effects, has gained substantial momentum. Plant-based formulations, in particular, are regarded as a promising avenue due to their perceived safety and affordability. In this context, the primary objective of this study is to examine the protective effects of an ethanolic extracts obtained from *C. Proximus* and *P. crispum* seeds, along with GA, and its nanogel emulsion, against urolithiasis induced in rats by EG and AC.

The evaluation of this study encompasses several facets, including an assessment of the mitigating effects on the antioxidant machinery within renal tissue, specifically catalase (CAT), reduced glutathione (GSH), and Malondialdehyde (MDA) levels. Additionally, the study examines serum toxicity markers, encompassing nitrogenous waste products such as blood urea nitrogen (BUN), uric acid, urea, and creatinine. Mineral levels, including calcium (Ca), magnesium (Mg), phosphorus (P), sodium (Na), and potassium (K), are also scrutinized. Furthermore, urinary markers, including urine pH, urine volume, creatinine clearance, uric acid, Ca, Mg, P, Na, K, and oxalate, are part of the assessment. The methodology extends to microscopic examination of urine, histopathological examination of kidney tissue, and immunohistochemistry examination. These comprehensive evaluations aim to provide a comprehensive understanding of the protective effects of *C. proximus* and *P. crispum* seeds ethanolic extracts/GA emulsion and its nanogel emulsion form, against EG and AC-induced urolithiasis in rat models.

## Materials and methods

### Materials

#### Chemicals and plant materials

Ethylene glycol and ammonium chloride were purchased from Sigma Co. (Poole, Dorset, UK); medium-viscosity sodium alginate (3500 cps); calcium chloride CaCl_2_ was supplied from Merck Chemicals Co. and was used as the crosslinker; polysorbate 80 Fenton Chemicals. ENDORE, MP.

*Cymbopogon proximus*, *Petroselinum crispum* seeds, and GA were purchased from the local market in Giza, Egypt.

#### Animals, diet and ethical statement

Twenty-four male Sprague Dawley (SD) rats weighing between 182 and 200 g (3–4 months) were sourced from the Animal House at the Veterinary Medicine faculty in Egypt. Stringently adhering to the National Institutes of Health Guide for the Care and Use of Laboratory Animals (Publication No. 85 − 23, revised 1985), all procedures and protocols concerning the care and handling of the animals were followed. Furthermore, ethical clearance for the present study was granted by the ethics committee of Veterinary Medicine at Cairo University, Egypt (ethical approval number: [Vet CU 09092023754]. Upon arrival, the rats underwent a one-week acclimatization period before being sorted into four groups (n = 6 per group). Each rat was housed in an individual polypropylene cage, ensuring optimal living conditions. Environmental parameters were meticulously regulated, maintaining a controlled temperature range of 24 ± 2 °C and relative humidity levels between 40% and 60%. The rats were subjected to a consistent 12-hour light-dark cycle and were allowed unrestricted access to both food and water throughout the entire duration of the study.

The diet employed in this study adhered to the AIN-93 diet guidelines [23], ensuring a well-balanced nutritional composition. The diet consisted of specific components: 12% of the total composition derived from casein as a protein source, 10% from corn oil, 10% from sucrose, 58.5% from maize starch, 5% cellulose, 3.5% from a salt mixture, and 1% from a vitamin mixture. Both the salt and vitamin mixtures were meticulously formulated in accordance with the AIN-93 guidelines [23].

### Methods

#### Preparations and characterization of emulsion and nanogel emulsion

##### Preparation of crude ethanol extracts of *C. Proximus* and *P. Crispum* seeds

The air-dried aerial parts of *C. proximus* and *P. crispum* seeds were initially cleaned and subsequently ground into a course powder using an electric grinder. Fifty grams (50 g) of each plant powder was soaked and stirred with an electric blender separately in 200 ml of ethyl alcohol 80% at a ratio of 1:4 (powder/solvent). Then, it was transferred into an airtight plastic container and kept for at least 48 h. Then, the mixture was filtrated and the solvent was removed using a vacuum rotary evaporator and the extract will be obtained [24]. The produced extract will be saved in a refrigerator at 4 ^o^C to avoid any contaminations until the time of use.

##### Preparation of an emulsion using ethanolic extracts of *C. Proximus* and *P. Crispum* seeds with GA

To prepare an emulsion from *P. crispum* seeds and *C. proximus* ethanolic extracts with GA, 4 g of GA powder were dissolved in distilled water at a concentration of wt/v %. Then the GA solution was blended with *P. crispum* seeds and *C. proximus* extracts with blend ratio of 1:1:1. The obtained emulsion was stirred for 2 h using a magnetic stirrer until completely mixed.

##### Phytochemical analysis using gas chromatography-mass spectrometry (GC/MS)

The phytochemical analysis of the ethanolic extracts from C. *proximus* with *P. crispum* seeds was conducted via GC/MS. The analysis was performed using a fused silica capillary column (30 m, 0.251 mm, 0.1 mm film thickness) with a Thermo Scientific Trace GC Ultra/ISQ Single Quadrupole MS, TG-5MS. Helium gas was utilized as the carrier gas at a steady flow rate of 1 mL/min in an electron ionization device with an ionization energy of 70 eV for GC/MS detection. The temperature of the injector and MS transfer line was adjusted to 280 ^o^C. The oven was set to start at 45 °C and hold it for two minutes. It was then programmed to increase to 150 °C at a pace of 7 °C per minute. After that, it was set to increase to 270 °C at a rate of 5 °C per minute (hold it for two minutes) and finally to 310 °C as the final temperature at a rate of 3.5 °C per minute (hold for 10 min). A percent relative peak area was used to evaluate the quantification of all the components that were discovered. Based on a comparison of the compounds’ relative retention times and mass spectra with the NIST, and WILLY library data of the GC/MS instrument, a preliminary identification of the compounds was carried out.

##### Preparation of a nanogel emulsion using ethanolic extracts of *C. Proximus* and *P. Crispum* seeds with GA

**Fig. 1:**
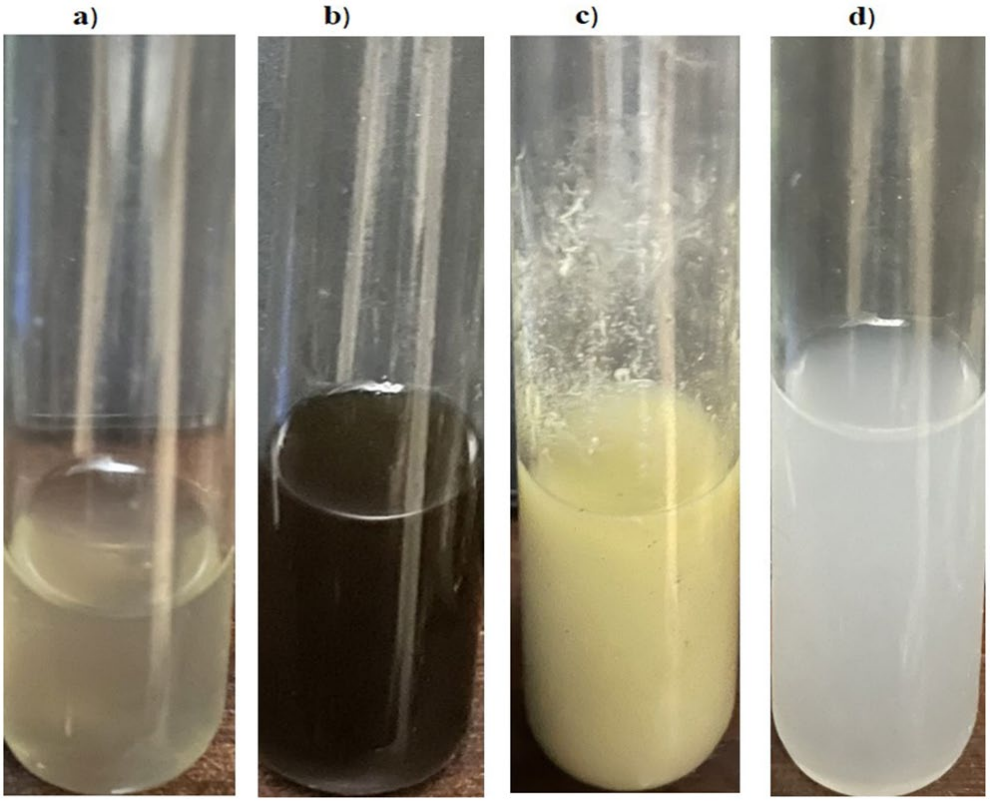
Macroscopic studies of (**a**) GA, (**b**) *C. proximus* and *P. crispum* seeds extracts, (**c**) *C. proximus* and *P. crispum* seeds extracts/GA emulsion, (**d**) *C. proximus* and *P. crispum* seeds/GA nanogel emulsion

The methodology of Sundararajan et al. [25] was modified to prepare nanogel emulsions using concentrated extracts of *C. proximus* and parsley seeds. Nanogel emulsion quantification was developed using a low-energy method. GA (4% wt./v) was prepared by dissolving the required amount in deionized water and stirred by a magnetic stirrer (800 rpm/1 h). Also, 2% (wt./v) sodium alginate was prepared, added to the GA solution, and stirred for 1 h until completely dissolving. In addition, the two extracts were added in the same concentration ratio. Four grams of each extract were dissolved in fifty milliliters of 50% v/v ethanol to create the extract solution. 2% polysorbate 80 was then added, and the mixture was stirred. To the previously prepared coarse extract polysorbate the resultant aqueous GA alginate solution was added dropwise at a flow rate of 1 mL/min. 7.5 ml of 18 Mm of aqueous CaCl_2_ solution was added dropwise (140 ml/30 sec) to the gum Arabic alginate solution under gentle stirring before addition to the extract polysorbate solution. The mixture was agitated for ten hours at 1200 rpm. After that, the nano gel emulsion was maintained at room temperature (20 °C), and its stability was assessed [26]. Figure 1(a and b) shows a macroscopic study of the prepared gum Arabic solution and the two-extract mixture respectively. Furthermore, Fig. 1 (c and d) shows the difference between the prepared micro and nano emulsions of GA, *C. proximus* and *P. crispum* seed extracts.

##### Particle size analysis and zeta potential measurement

The average size distribution and zeta potential of the GA loaded with *C. proximus* and *P. crispum* seed extracts nanogel emulsion were determined by dynamic light scattering (DLS) with a Zeta Sizer (Malvern Instruments, UK) and a Zeta Potential analyzer (Nicomp 380 ZLS, USA), respectively. Measurements were made on the aqueous suspension of the NPs. The emulsion sample (2 drops) was diluted in 2 mL of water and put into a cuvette to measure the particle size. A capillary cell (25 µL) held the sample for the zeta potential measurement, which was subsequently diluted in two milliliters of water. The Stokes-Einstein relation and its corresponding polydispersity index (PDI) were used to calculate the particle size as a Z-average. Zeta potential and particle size were measured three times for every sample.

#### Design of the experimental study

##### Induction of urolithiasis

The evaluation of antiurolithiatic activity was conducted using a urolithiasis animal model induced by the simultaneous administration of ethylene glycol and ammonium chloride in male SD rats. This experimental approach is as per earlier reported methods, with some modifications.

In a 14-day protocol involving the provision of EG at a concentration of 0.75% v/v and AC at a concentration of 1% w/v supplied through drinking water ad libitum access, this induction protocol aimed to induce urolithiasis, thereby stimulating the formation of calcium oxalate crystals [27].

##### Preventive study model: dosing and grouping

The rats were divided into four groups (n = 6) and orally administered either the emulsion or nanogel emulsion using an oral tube. Throughout the experimental study, all rats had ad libitum access to both drinking water and diet.

- ***Group 1***: In the normal control group, rats had ad libitum access to drinking water for 21 days.
- ***Group 2***: Urolithiasis group: rats had unrestricted access to drinking water for 21 days. Starting from day 8 and continuing until day 21, they were given drinking water containing 0.75% EG V/V along with 1% AC W/V to induce urolithiasis.
- ***Group 3***: Emulsion group, rats were orally administered *C. proximus* and *P. crispum* seeds ethanolic extracts/GA emulsion (600 mg/kg b.w./day) for 21 days. From day 8 to day 21, they received a dose of 0.75% EG V/V + 1% AC in their drinking water.
- ***Group 4***: Nanogel emulsion group: rats were orally administered *C. proximus* and parsley seeds ethanolic extracts/GA nanogel emulsion (600 mg/kg b.w./day) for 21 days. From day 8 to day 21, they received a dosage of 0.75% EG V/V + 1% AC in their drinking water.

**Fig. 2:**
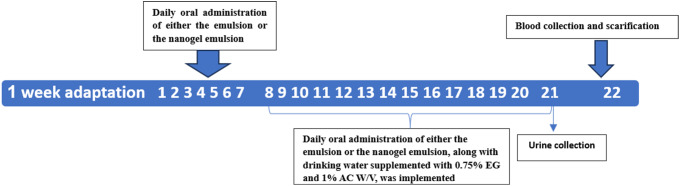
Illustrates a schematic diagram depicting the experimental design for inducing urolithiasis in rats through EG and AC in drinking water for 14 days. Daily oral administration of either the emulsion (600 mg/kg) and nanogel emulsion (600 mg/kg) was conducted for 21 days. Following the last treatment, urine samples were collected from each rat. Twenty-four hours later (day 22), blood samples were obtained. Finally, the rats were sacrificed for kidney harvesting

##### Body weight and food intake measurement

During the study period, all groups of rats received a balanced diet. Weekly records were kept for each rat, documenting their body weight and food intake. At the end of the study, we calculated the total food intake, body weight gain, and feed efficiency ratio.

##### Collection and assessment of urine samples

Individual metabolic cages were assigned to each rat for the collection of 24-hour urine samples on the 21st day of evaluation, the rats had unrestricted access to drinking water. Following the urine collection, immediate assessments were made for urine pH using pH test strips (Macherey-Nagel GmbH & Co. KG, Düren, Germany) and urine volume. A drop of urine was dispensed onto a clean glass slide and examined under a light microscope (Olympus microscope, model CX41, Japan). Photomicrographs were captured for each urine sample. The micrographs were subsequently employed to estimate the quantity of crystals present. The grading system for the severity of crystalluria was as follows: “-” indicating no crystals observed; “+” denoting 1 to 5 crystals per high power field (x400); “++” indicating 6 to 20 crystals per high power field; and “+++” indicating more than 20 crystals per high power field.

The 24-hour urine samples were divided into two halves and stored at -20 °C. Prior to storage, one half was acidified with 1–2 drops of 5 M HCl, while the other remained non-acidified. Urine parameters including urea (Fawcett and Scott,1960) [28], creatinine (Larsen, 1972) [29], uric acid (Barham and Trinder, 2009) [30], total protein (Rheinhold, 1953) [31], albumin (Doumas et al., 1971) [32], were analyzed from the non-acidified urine samples. The acidified sample was employed for the assessment of urinary biochemical parameters such as calcium (Kessler and Wolfman) [33], phosphorus (El-merzabani et al.) [34], potassium (Sunderman) [35], sodium (Henry et al.) [36], and magnesium [37], all of which were colorimetrically assayed using standard commercial kits acquired from Salucea Co., Netherlands. These procedures were conducted in accordance with the manufacturer’s instructions. Urinary oxalate levels were determined using a quantitative rat oxalate colorimetric assay kit (Elabscience Biotechnology Co., Ltd., China, catalog No. E-BC-K892-M) at a wavelength of 550 nm for urinary oxalate analysis, following the manufacturer’s instructions.

##### Blood sampling and kidney collection

Twenty-four hours after the final treatment, each rat underwent anesthesia, administered as a combination of ketamine (100 mg/kg) and xylazine (10 mg/kg) following an overnight fasting period. Approximately 2 ml of blood was then drawn from the retro-orbital venous plexus of the rats’ eyes and collected in clear test tubes. Subsequently, the blood samples underwent centrifugation at 3000 rpm/min at 4 °C for 10 min using a laboratory centrifuge (2k15, Sigma, Germany). The resulting sera were stored at -20 °C until further analysis of the biochemical parameters.

Following euthanasia by cervical dislocation, their kidney specimens were promptly excised, immersed in a cold saline solution (0.9% NaCl), and gently blotted dry using filter paper. The specimens were then weighed.

The left kidney specimens, fixed in 10% neutral buffered formalin, were prepared for histopathological and immunohistochemistry examinations. Simultaneously, the right kidney specimens were immersed in cold phosphate buffer (0.1 M; pH 7.4) to create a 20% homogenate using a tissue homogenizer (MPW-120, BitLab Medical Instruments, Poland). This homogenization process was followed by centrifugation at 4000 rpm/min for 10 min at 4 °C. The resulting supernatant was carefully collected and stored at -80 °C for subsequent measurement of CAT, GSH, and MDA levels. All experimental procedures strictly adhered to approved ethical guidelines.

The kidney index was calculated using the following formula: kidney index = (kidney weight/body weight) ×100.

##### Assessment of serum and renal biomarkers

Serum kidney function indices, including urea (Fawcett and Scott,1960) [28], creatinine (Larsen, 1972) [29], uric acid (Barham and Trinder, 2009) [30], total protein (Rheinhold, 1953) [31], albumin (Doumas et al., 1971) [32], calcium (Kessler and Wolfman, 1964) [33], phosphorus (El-merzabani et al., 1977) [34], potassium (Sunderman, 1958) [35], and sodium (Henry et al., 74) [36], were assessed using commercial kits from Salucea Co., Netherlands. MDA levels were determined according to the method of Satoh, 2022 [38], while CAT and GSH were analyzed following the method outlined by Luck, 1978 and Sedlak & Lindsay, 1968 [39, 40], respectively. These analyses utilized a commercially available colorimetric kit (Biodiagnostics, Egypt; catalog no. MD 25 29, CA 25 17, and GR 25 11) according to the manufacturer’s instructions. Optical density measurements for all parameters were conducted using a spectrophotometer (Shimadzu UV-2401 PC, Australia).

##### A. Histopathological and immunohistochemical evaluations

The kidney specimens, preserved in formalin, underwent a standard processing procedure involving dehydration in varying alcohol concentrations, clearance in xylol, and embedding in paraffin. Subsequently, thin sections measuring 4–5 μm were obtained from the paraffin blocks, followed by staining using Hematoxylin and Eosin (H&E) [41].

For immunohistochemical assessment, the paraffin sections underwent antigen retrieval through microwave heating for 25 min at 720 W. Post-retrieval, they were incubated overnight at 4 °C with primary antibodies: rabbit monoclonal anti-rat caspase-3 (1:1000 dilution, Abcam, ab184787, Cambridge, MA, USA), and mouse monoclonal anti-rat TNF-α (1:500 dilution, Abcam, ab220210, Cambridge, MA, USA). Following PBS washing, sections were then incubated at room temperature for 30 min with respective biotinylated secondary antibodies at a 1:200 dilution (Dako Corp.), and streptavidin/ALP alkaline phosphatase complex, also at a 1:200 dilution (Dako Corp.). Visualization of antibody binding sites was accomplished using DAB (Sigma), followed by PBS washing and counterstaining with Hematoxylin for 2–3 min. The final steps involved sample dehydration in escalating ethanol solutions, double xylene soaking for 5 min each at room temperature, mounting, and examination under a high-power light microscope [42].

Quantification of marker expression’s positive brown area was conducted by measuring the percentage area across seven high-power microscopic fields using Image analysis software (Image J, 1.46a, NIH, USA).

##### B. Renal crystal deposition

The evaluation of renal crystal deposits followed a graded scale: zero indicated the absence of crystal deposits, 1 denoted the presence of crystal deposits at the papillary tip, 2 indicated crystal deposits at the cortico-medullary junction, and 3 signaled crystal deposits in the cortex. In instances where crystals were found in multiple locations, the scores were aggregated to derive a comprehensive final score [43].

#### Statistical analysis

Statistical analyses were conducted using SPSS version 25. The results obtained from the animal experiment were expressed as mean ± standard error (SE) and subjected to statistical scrutiny using one-way analysis of variance (ANOVA), followed by the Duncan test for post hoc analysis. A significance level of P ≤ 0.05 was applied to ascertain statistical significance. In instances where frequency data were involved, the Kruskal-Wallis H test, a nonparametric analysis, was employed, followed by the Mann-Whitney U test for further assessment. Median values were reported for the nonparametric data.

## Results

### Phytochemicals identified by GC/MS analysis

The dried ethanolic extracts of *C. proximus* and *P. crispum* seeds underwent GC-MS analysis to identify potential bioactive compounds. The analysis revealed a diverse array of phytochemicals, indicating that the ethanolic extract of *C. proximus* with *P. crispum* seeds contained twenty compounds, totaling 100% based on area percentage. Among the most dominant compounds were Lutein, carotenes, Fucoxanthin, Milbemycin b, phenylporphyrin, and other phenyl derivative compounds (Table 1).

**Table 1:** Compounds identified by GC-MS in the extract of *C. proximus* and *P. crispum* seeds

Peak	Name	Formula	R_t_ (min)	Area %
1	“á,.Psi.-Carotene,3’,4’-didehydro-1’,2’-dihydro-1’,2’-dihydroxy-,(2’R)”	C40H56O2	7.32	5.16
2	“Dodecachloroperylene”	C20Cl12	7.62	4.98
3	“2,2-Bis[4-[(4,6-dichloro-1,3,5-triazin-2 yl)oxy]phenyl]-1,1,1,3,3,3-hexafluoropropane”	C21H8Cl4F6N6O2	8.83	5.74
4	“(22R)-6á,11á,21-Trihydroxy-16à,17à-propylm ethylenedioxypregna-1,4-diene-3,20-dione”	C25H34O7	10.17	4.97
5	5,11,17, 23-tetrakis(1,1-dimethylethyl)-28 methoxypentacyclo[19.3.1.1(3,7)0.1(9,13)0.1(15,19)]octacosa-1(25),3,5,7(28),9,11,13 (27),15,17,19(26),21,23-dodecene-25,26,27-triol	C45H58O4	10.87	5.69
6	Vitamin B12	C63H88CoN14O14P	11.73	4.62
7	“3-Hydroxy-1-(4-{13-[4-(3-hydroxy-3-phenylacryloyl)phenyl]tridecyl}-phenyl)-3-phenylprop-2-en-1-one”	C43H48O4	14.72	3.76
8	Fucoxanthin	C42H58O6	15.84	4.82
9	“Milbemycin b,13-chloro-5-demethoxy-28-deoxy-6,28-epoxy-5-(hydroxyimino)-25-(1-methylethyl)-, (6R,13R,25R)”	C33H46ClNO7	18.88	4.15
10	2-Butyl-5,10,15,20-tetraphenylporphyrin	C48H38N4	23.12	4.95
11	“ë-chloro-2,4-bis(2-chloroethyl)-6,7-bis[2-(meth oxycarbonyl)ethyl]-1,3,5-trimethylporphyrin”	C35H37Cl3N4O4	40.67	8.26
12	Lutein	C40H56O2	46.72	3.85
13	“(Octamethoxy-tetraphenyl)-resorcin [4]arene diamond conformation”	C44H56O8	48.02	4.36
14	“2,2-Bis[4-[(4,6-dichloro-1,3,5-triazin-2-yl)oxy ]phenyl]-1,1,1,3,3,3-hexafluoropropane”	C21H8Cl4F6N6O2	48.14	5.53
15	Tetrahydrocannabinolcarbonic	C28H19D9F12O5	49.27	4.23
16	Nephthoside − 1,2’,3’,4’-Tetraacetate	C40H56O10	49.54	5.00
17	“2,4,6,8,10-Tetradecapentaenoic acid,9a-(acetyloxy)-1a,1b,4,4a,5,7a,7b,8,9,9a-decahydro-4a,7b-dihydroxy-3-(hydroxymethyl)-1,1,6,8-tetramethyl-5-oxo-1 H-cyclopropa [3, 4]benz[1,2-e]azulen-9-yl ester,[1aR-(1aà,1bá,4aá,7aà,7bà,8à,9á,9aà)]-”	C36H46O8	49.71	6.28
18	“3-Desoxo-3,16-dihydroxy-12-desoxyphorbol 3,13,16,20-tetraacetate”	C28H38O10	52.79	4.87
19	TRANS-2-PHENYL-1,3-DIOXOLANE-4-ME THYL	C28H40O4	53.06	3.89
20	“2,6-Bis(2,3,5-triphenyl-4-oxocyclopentadienyl) pyridine”	C51H33NO2	54.59	4.89
	Total			100

### Particle size distribution and zeta potential

**Fig. 3:**
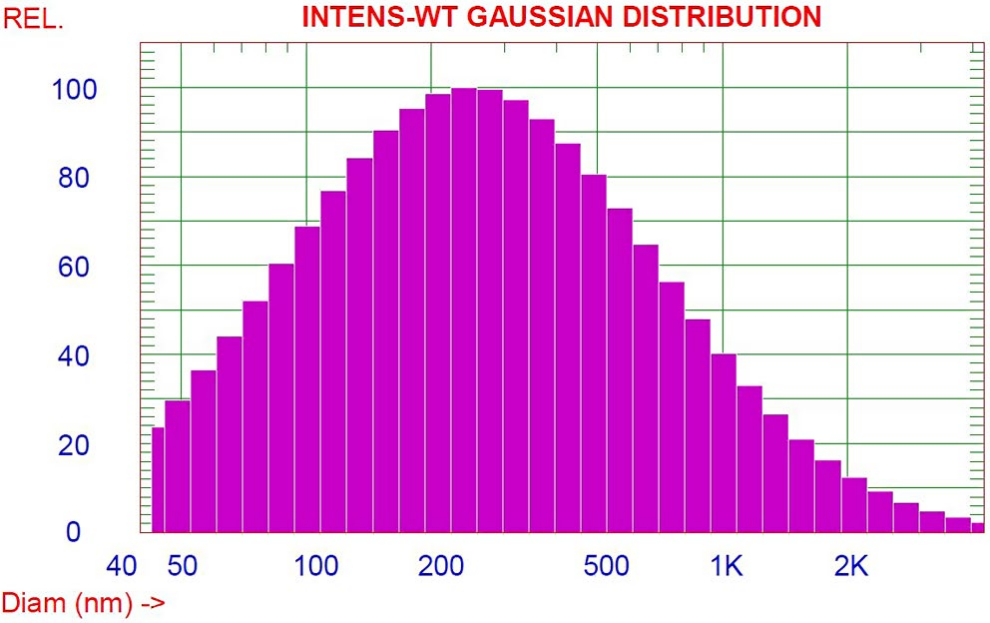
DLS size distribution curve of *C. proximus* and P. *crispum* seed extract/GA nanogel emulsion

The mean diameter and the average particle size distribution of *C. proximus* and *P. crispum* seed extracts/GA nanoparticles were determined by Dynamic Light Scattering (DLS) and the obtained data was illustrated in Fig. 3. The prepared nanogel emulsion particles have a mean diameter of 425 ± 1.039. It is obvious from the figure that 25% of the prepared nanoparticles have an average diameter < 141.1 nm, 50% have an average diameter < 266.7 nm and 75% have an average diameter < 520. The prepared nanogel emulsion particles are within the nanometer size range and have a large external surface area that improves their efficacy and their interaction with the medium in which they are dispersed.

**Fig. 4:**
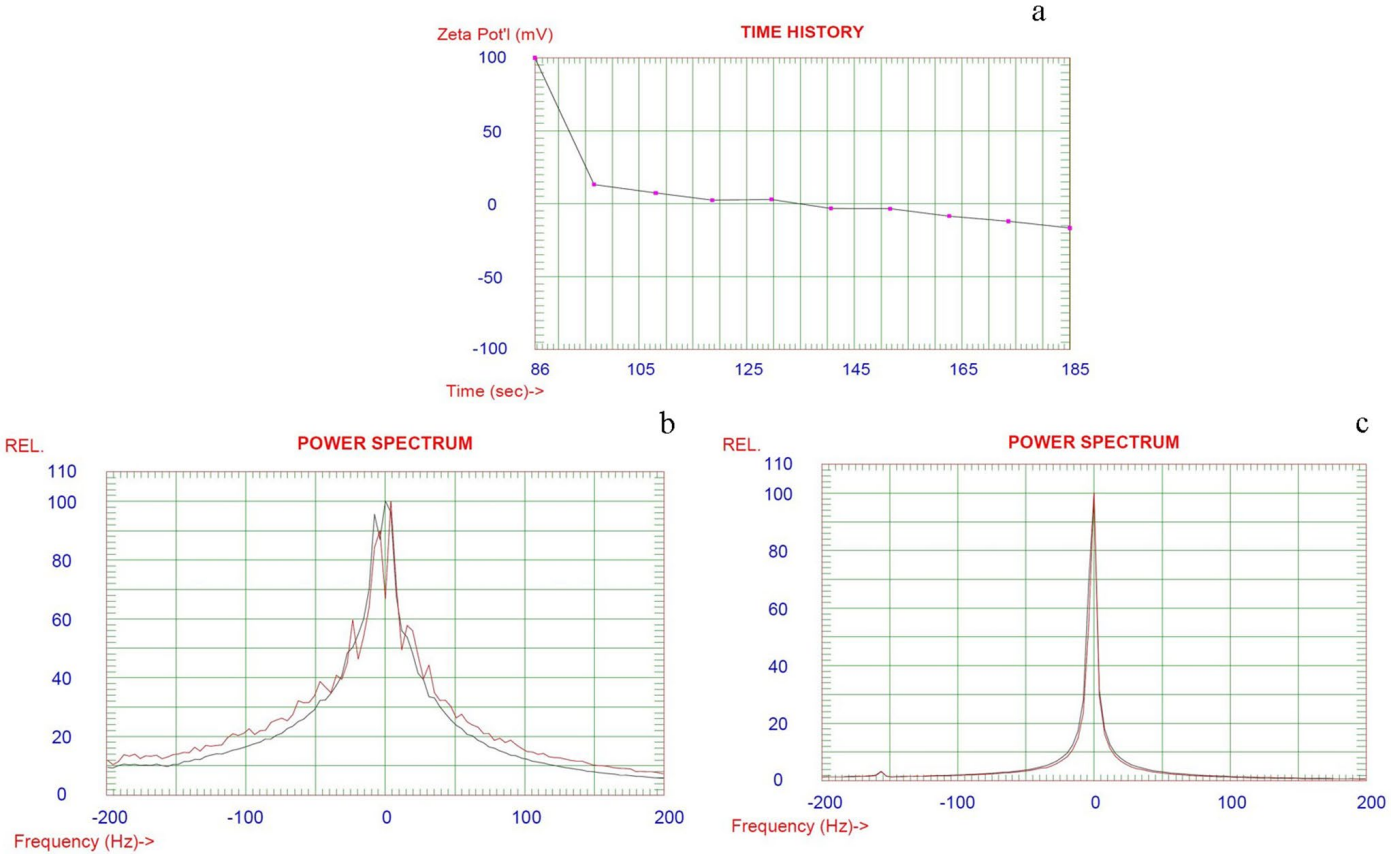
Zeta potential in mV of (**a**) GA (**b**) *C. Proximus* and *P. crispum* seeds extracts/GA and (**c**) *C. proximus* and *P. crispum* seeds extracts/ GA nanogel emulsion

Furthermore, the Zeta potential of GA solution, *C. proximus* and *P. crispum* seeds extracts/GA emulsion, and *C. Proximus* and *P. crispum* seeds extracts/GA nanogel emulsion were measured and illustrated in Fig. 4 (a, b and c). It is clear from the figure that the value of GA solution was − 16. This electronegativity is due to superficial hydroxyl groups in its backbone structure, which gave a − 27.31 mV overall surface charge. Zeta potential value increased to -23.36 mV when the extract was added to GA solution but increased greatly, as shown in Fig. 4 (c) in the case of GA nanogel emulsion which reached − 67.5 mV, which indicates higher stability. This high zeta potential value is a confirmation of the good stability of the prepared nanogel emulsion formulation. As a result, the prepared GA nanogel emulsion has better stability and can be used in drug delivery systems and food nanotechnology techniques.

### Body weight and nutritional parameters

The nutritional parameters for each experimental group are detailed in Table (2). The results depict notable differences between the control group and the model group. In the model group, a significant reduction in body weight gain and feed efficiency ratio were observed, accompanied by an increase in relative kidney weight (p < 0.05). However, upon comparing the model group to both treated groups, it is evident that disease progression was effectively restrained. Remarkably, both treated groups exhibited a significant elevation in feed efficiency ratio and body weight gain, coupled with a marked reduction in relative kidney weight (p < 0.05).

**Table 2:** Body and kidney weight and nutritional indices of the studied experimental rat groups

Parameters	Group 1	Group 2	Group 3	Group 4
Initial weight (g)	191.2 ± 2.9^**a**^	191.16 ± 2.8^**a**^	191.16 ± 2.6^**a**^	191.16 ± 2.6^**a**^
Final weight (g)	221.10 ± 6.43^**a**^	199.43 ± 4.49^**b**^	212.26 ± 5.56^**a**^	216.65 ± 4.17^**a**^
Body weight gain (g)	29.90 ± 7.23^**a**^	8.26 ± 4.16^**b**^	21.10 ± 6.11^**a**^	25.48 ± 5.36^**a**^
Food intake (g)	313.33 ± 4.42^**a**^	297.35 ± 4.50^**b**^	302.17 ± 4.75^**a**^	305.83 ± 3.82^**a**^
Feed efficiency ratio	0.095 ± 0.02^**a**^	0.027 ± 0.02^**b**^	0.06 ± 0.03^**a**^	0.08 ± 0.04^**a**^
kidney weight (g)	1.32 ± 0.04^**a**^	2.20 ± 0.05^**b**^	2.24 ± 0.11^**b**^	2.04 ± 0.09^**b**^
kidney index	0.59 ± 0.01^**a**^	1.10 ± 0.02^**b**^	1.01 ± 0.06^**b**^	0.94 ± 0.05^**c**^

In each row, identical letters indicate non-significant difference, while different letters signify a significant difference at p < 0.05. The data are presented as mean values ± standard error, with 6 rats in each group. Feed efficiency= (body gain/total food intake)

### Urine analysis

**Fig. 5:**
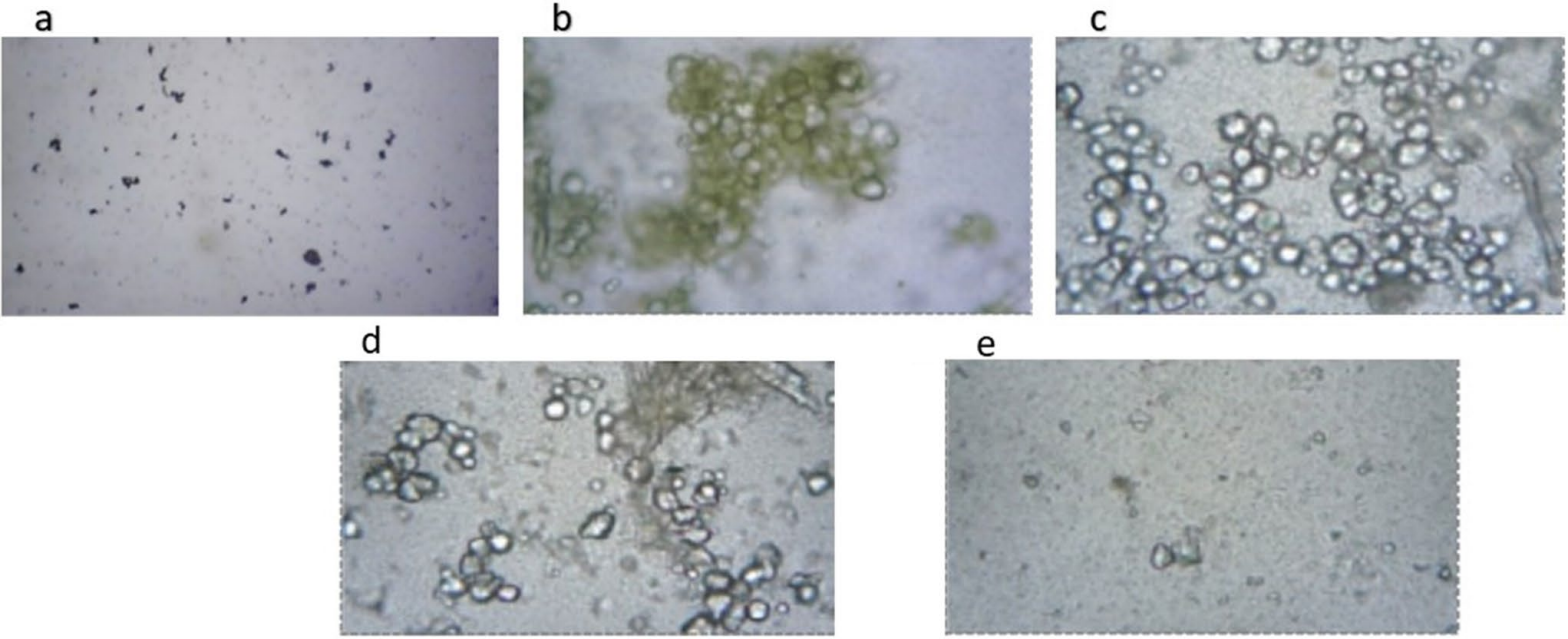
Microscopic analysis of urine among various experimental groups; **A** droplet of urine is applied onto a glass slide and examined under an optical light microscope to quantify the number of calcium oxalate crystals. **a**) Group 1, Demonstrates absence of oxalate crystals, **b** & **c**) Group 2, Indicates a high presence of oxalate crystals (+++), **d**) Group 3, Exhibits a moderate count of oxalate crystals (“++”)., **e**) Group 4, Shows a lower count of oxalate crystals (“+”)

The urolithiatic rats displayed a significant decrease in urine volume and PH. Treatment with either the emulsion or nanogel emulsion resulted in a noteworthy increase in these parameters. Urine samples from the normal control group showed an absence (“-“) of oxalate crystals. Conversely, the urine of urolithiatic rats exhibited a significant presence of oxalate crystals (“+++”). Among urolithiatic rats treated with the emulsion, a moderate number of oxalate crystals was observed (“++”). Conversely, urolithiatic rats treated with nanogel emulsion displayed fewer oxalate crystals (“+”), as depicted in Fig. 5.

Moreover, the urolithiatic control group demonstrated a significant increase in urolithiasis-promoting factors, such as the urinary excretion of calcium, phosphate, oxalate, and uric acid. Moreover, urolithiatic animals displayed a notable decrease in the excretion of magnesium, alongside a decline in renal function, as evidenced by a significant decrease in creatinine clearance, creatinine levels, and sodium levels, coupled with an increase in urea, potassium, total protein, and albumin, in comparison to normal control animals.

The treatment with emulsion or nanogel emulsion resulted in a significant reduction in urolithiasis-promoting factors, including excreted calcium, oxalate, phosphate, and uric acid. Additionally, kidney function showed marked improvement following treatment, characterized by enhancements in creatinine clearance, creatubinine levels, total protein, albumin levels, sodium levels, and potassium levels when compared to urolithiatic control rats. The most significant improvements were observed in the nanogel emulsion group. These findings underscore the beneficial effects of nanogel emulsion in mitigating urolithiasis-related parameters and improving kidney function (Table 3).

**Table 3:** Depicts the impact of oral administration of emulsion and nanogel emulsion on urine analysis parameters, specifically magnesium, citrate, and uric acid, within the studied experimental groups

Parameters	Group 1	Group 2	Group 3	Group 4
Urine volume	19.2 ± 1.34 ^a^	10.66 ± 0.80 ^b^	17.5 ± 1.18 ^a^	19.50 ± 2.81 ^a^
PH	6.74 ± 0.16 ^a^	5.75 ± 0.25 ^b^	6.60 ± 0.24 ^a^	6.75 ± 0.25 ^a^
Creatinine mg/24 h	37.68 ± 1.08 ^a^	13.71 ± 2.30 ^b^	25.12 ± 1.49^c^	32.15 ± 0.15 ^a^
Creatinine clearance ml/minute	0.62 ± 0.04 ^a^	0.033 ± 0.01 ^b^	0.17 ± 0.30 ^c^	0.32 ± 0.05^d^
Urea mg/24 h	3.61 ± 0.88 ^a^	18.83 ± 0.76 ^b^	10.67 ± 0.42 ^c^	5.99 ± 0.30 ^d^
Uric acid mg/24 h	2.63 ± 0.34 ^a^	10.05 ± 0.72 ^b^	6.30 ± 0.47 ^c^	3.61 ± 0.20 ^a^
Total protein g/24 h	0.27 ± 0.44 ^a^	1.09 ± 0.51 ^b^	0.62 ± 0.08 ^c^	0.43 ± 0.06 ^a^
Albumin g/24 h	0.048 ± 0.02 ^a^	0.11 ± 0.03 ^b^	0.046 ± 0.01 ^a^	0.050 ± 0.02 ^a^
Calcium mg/24 h	3.48 ± 0.37 ^a^	9.81 ± 1.48 ^b^	6.79 ± 0.30 ^a^	4.49 ± 0.34 ^a^
Oxalate mg/24 h	0.52 ± 0.2 ^a^	1.46 ± 0.11 ^b^	0.93 ± 0.08 ^c^	0.81 ± 0.10 ^ac^
Phosphate mg/24 h	5.78 ± 1.06 ^a^	18.42 ± 1.81 ^b^	11.64 ± 0.84 ^c^	8.65 ± 0.48 ^ac^
Magnesium mg/24 h	4.53 ± 0.59 ^a^	1.73 ± 0.79 ^b^	2.86 ± 1.13 ^c^	3.76 ± 1.01 ^a^
Sodium mmol/24 h	57.52 ± 5.52 ^a^	32.57 ± 4.8 ^b^	46.72 ± 1.43 ^a^	51.66 ± 1.38 ^a^
Potassium mmol/24 h	25.70 ± 3.50 ^a^	35.00 ± 1.95 ^b^	29.35 ± 0.39 ^a^	27.84 ± 0.80 ^a^

In each row, identical letters indicate non-significant difference, while different letters signify a significant difference at p < 0.05. The data are presented as mean values ± standard error, with 6 rats in each group. Feed efficiency= (body gain/total food intake)

### Kidney function and renal oxidative stress biomarkers

EG and AC-induced urolithiasis in rats resulted in a significant exacerbation of various renal function parameters Table 4. This included elevated levels of serum creatinine, BUN, urea, uric acid, total protein, and albumin, as well as a noteworthy increase in calcium, phosphorus, and sodium, coupled with a reduction in serum potassium levels when compared to the normal control animal group.

**Fig. 6:**
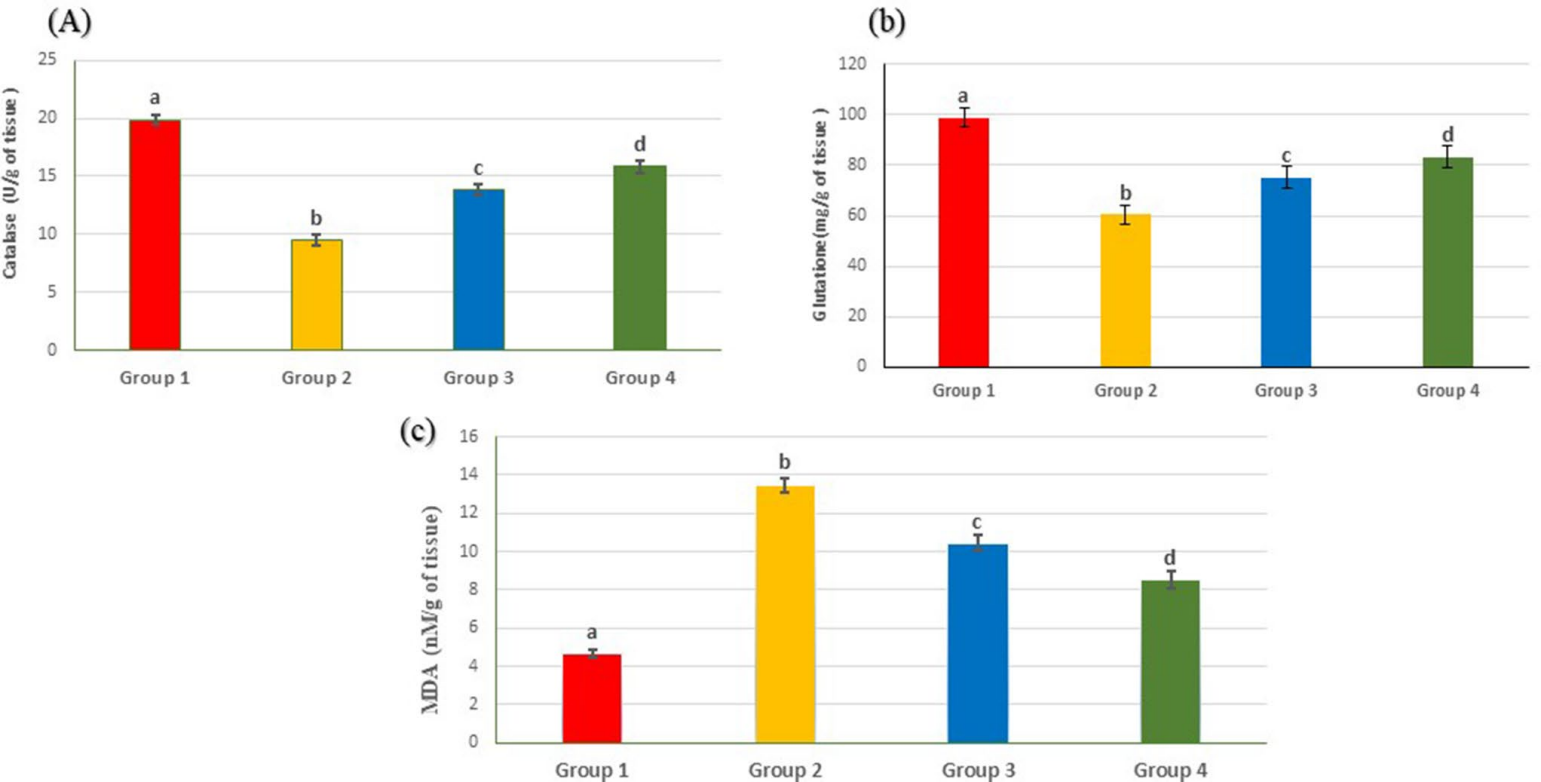
Illustrate the impact of oral administration of emulsion and nanogel emulsion on renal oxidative stress markers in renal tissue across the different studied groups: Group 1 (negative control), Group 2 (urolithatic group), Group 3 (emulsion), and Group 4 (nanogel emulsion). (**A**) Renal catalase, (**B**) Renal glutathione, (**C**) Renal malondialdehyde. Values are presented as mean ± SEM (n = 6). Different letters within the columns indicate a significant difference at p < 0.05

Conversely, the co-administration of either emulsion or nanogel emulsion significantly attenuated serum concentrations of creatinine, urea, uric acid, and BUN, as well as total protein and albumin levels, when compared to urolithiatic rats. Particularly noteworthy improvements were observed in the nanogel emulsion. Moreover, urolithiatic rats displayed significantly higher levels of MDA and lower levels of glutathione and catalase activity in kidney tissue compared to the control groups. However, upon administration of either the emulsion or nanogel emulsion, there was a decrease in MDA levels and an increase in glutathione levels and catalase activity when compared to the urolithiatic animals, as depicted in Fig. 6. These results suggest that both the emulsion and nanogel emulsion hold potential as therapeutic agents against anti-urolithiasis, with the latter demonstrating superior efficacy.

**Table 4:** Illustrates the impact of oral administration of emulsion and nanogel emulsion on renal function and various parameters, encompassing calcium, phosphorus, sodium, and potassium within the studied experimental rat group

Parameters	Group 1	Group 2	Group 3	Group 4
Creatinine mg/dl	0.81 ± 0.18 ^a^	2.78 ± 0.20 ^b^	1.72 ± 0.16 ^c^	1.13 ± 0.11 ^a^
Urea mg/dl	17.08 ± 2.85 ^a^	85.30 ± 1.88 ^b^	36.53 ± 1.56 ^c^	22.71 ± 0.64 ^d^
BUN mg/dl	7.97 ± 1.33 ^a^	39.83 ± 0.88 ^b^	17.52 ± 0.72 ^c^	10.61 ± 0.30 ^d^
Uric acid mg/dl	1.60 ± 1.42 ^a^	4.71 ± 0.05 ^b^	2.50 ± 0.28 ^c^	1.97 ± 0.07 ^d^
Total protein g/dl	5.53 ± 0.27 ^a^	6.67 ± 0.32 ^b^	6.24 ± 0.15 ^b^	5.81 ± 0.24 ^a^
Albumin g/dl	3.13 ± 0.06 ^a^	4.71 ± 0.32 ^b^	3.81 ± 0.40 ^a^	2.94 ± 0.075 ^a^
Calcium mg/dl	11.40 ± 0.14 ^a^	20.32 ± 0.89 ^b^	15.34 ± 0.44 ^c^	12.19 ± 0.13 ^a^
Phosphorus mg/dl	4.44 ± 0.07 ^a^	14.92 ± 0.18 ^b^	9.42 ± 0.38 ^c^	5.10 ± 0.28 ^a^
sodium mmol/dl	140.24 ± 2.11 ^a^	157.57 ± 2.55 ^b^	149.03 ± 1.98 ^c^	144.39 ± 2.13 ^ac^
potassiummmol/dl	6.06 ± 0.11 ^a^	4.73 ± 0.26 ^b^	5.32 ± 0.69 ^ab^	5.81 ± 0.22^ab^

In each row, the use of the same letters indicates a non-significant difference, while different letters signify a significant difference at p < 0.05. The data is presented as mean values ± standard error for each group, with six rats in each group

### Histopathological evaluation

**Fig. 7:**
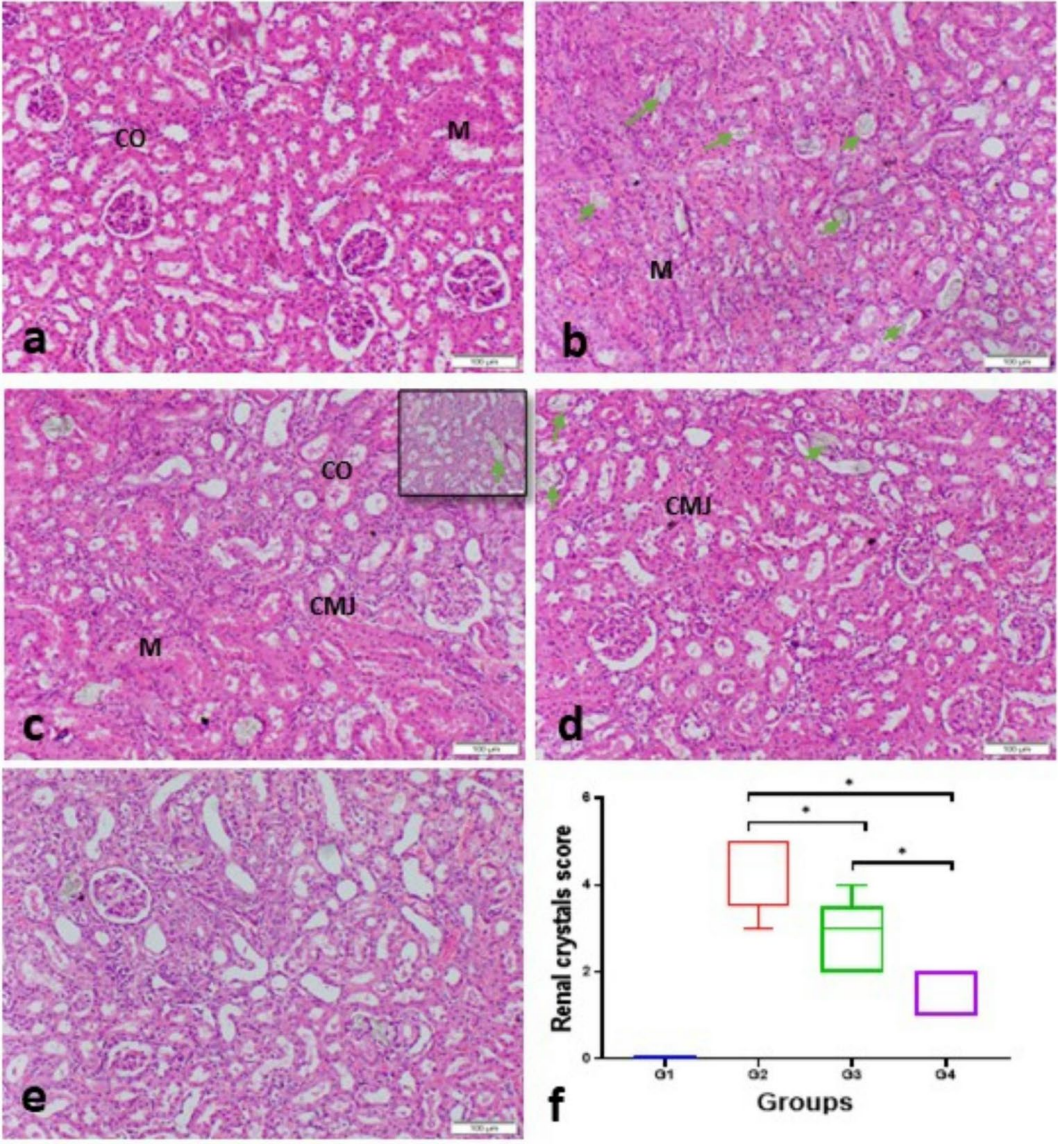
Illustrates representative photomicrographs displaying H & E stained renal tissue of rats, **a**) Negative Control (untreated rats), showing the normal tissue architecture of renal parenchyma, glomerulus and renal tubules, **b** & **c**) positive control showing diffuse renal crystal deposition (green arrows), congested blood vessels and diffuse interstitial nephritis with high magnification in upper right corner showing renal crystal deposition inside renal tubule, **d**) Group 3 (rats treated with emulsion) showing multifocal renal crystal deposition with moderate inflammatory cells infiltration, **e**) Group 4 (rats treated with nanogel emulsion) showing significant decrease in crestal deposition and mild inflammatory cells infiltration, **f**) Renal crystal score in different experimental groups represented as median. (scale bar 50 and 100 μm). CO: renal cortex, M; renal medulla and CMJ; cortico-medullary junction

The renal tissue of animals in group 1 was histologically normal (Fig. 7a), conversely, rats in group 2 displayed severe damage characterized by inflammatory infiltration, and focal necrosis in renal epithelial lining of renal tubules with renal tubule enlargement with crystal deposits in the lumen were observed. (Fig. 7b and c). Renal tissues from groups 3 and 4 showed improvements; as these groups included minor inflammatory infiltration and mild tubule dilation. (Fig. 7d and e). However, significant differences were found between groups 3 and 4 (Fig. 7f), with the fourth group exhibiting an amendment effect (p < 0.05). The renal crystal scores were significantly higher for groups 2 and 3 compared to group 4 (p < 0.05) (Fig. 7C).

### Immunohistochemical expression of cleaved caspase-3 and TNF-α

**Fig. 8:**
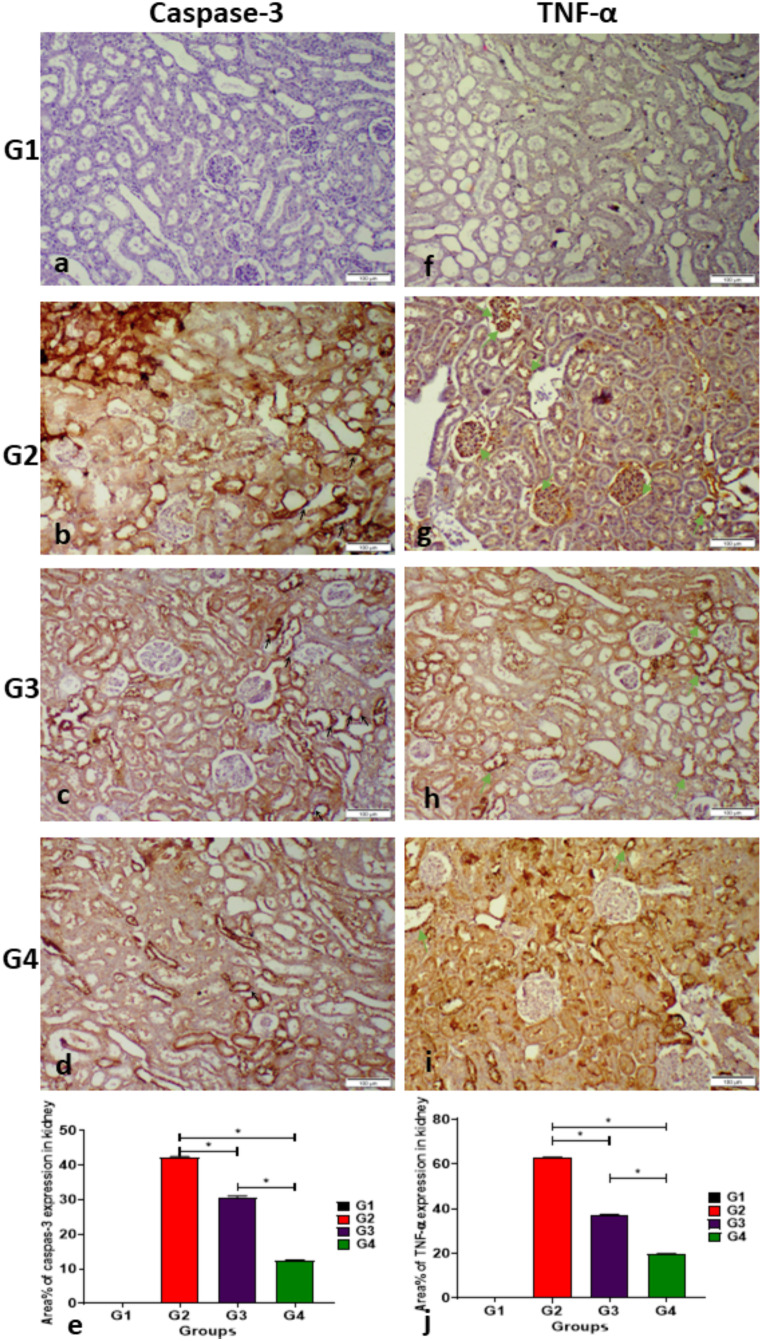
Immunohistochemical staining of cleaved (active) caspase-3 and TNF-α in renal tissues across different groups. Representative photomicrographs (scale bar 100 μm) of renal tissues exhibit brown immunostaining expression of caspase-3 (**a**-**d**) and TNF-α (**f**-**i**) as follows: (**a** and **f**) Show absence of staining in the negative control rats, (**b** and **g**) Demonstrate strong immune-expression in the positive control group, (**c** and **h**) Depict moderate expression in group 3, and (**d** and **i**) Reveal mild immune reactive cells in group 4. Furthermore, (**i** and **j**) indicate the immunostaining area (%) of (**i**) cleaved caspase-3 and (**j**) TNF-α expression in the renal tissue of rats treated with emulsion and nanogel emulsion. Data analysis used One-way analysis of variance (ANOVA) followed by the Duncan post-hoc test. *Significant differences were considered at p ≤ 0.05

Figure 8 depicts Caspase-3 and TNF-α in renal tissue sections. In summary, normal control rats exhibited negative expression for both immune markers (Fig. 8a, e). In contrast, group 2 (Fig. 2) demonstrated robust immune expression. Meanwhile. Sections from rats in group 3 exhibited a moderate immune response (Fig. 8d, h), whereas sections from rodents in group 4 displayed a mild immune response. Figure 8 (i, j) indicated the area % expressions of active caspase-3 and TNF-α in the renal tissue of rats across various experimental groups showed a significant elevation in immune expression compared to the negative control group, and the group 4 demonstrated a significant decrease in cleaved caspase-3 and TNF-α expression, as compared with group 2 and 3

## Discussion

Due to contemporary lifestyles, urolithiasis remains a pervasive global medical challenge, and its prevalence is steadily escalating, evidently manifesting a pronounced tendency towards high recurrence rates. However, the utilization of medicinal plants holds profound significance on a global scale, serving not only as standalone remedies but also as complementary adjuncts to conventional medicinal approaches [44, 45]. The diverse components of medicinal plants, including seeds, leaves, flowers, fruits, stems, and roots, have consistently emerged as reservoirs of bioactive compounds [46, 47].

The ingestion of ethylene glycol by rats has long been a pivotal experimental model for investigating nephrolithiasis. However, the deposition of kidney crystals following ethylene glycol administration can be inconsistent. To consistently induce high rates of kidney crystal deposition, researchers have employed the combination of ammonium chloride with ethylene glycol. When male SD rats were subjected to treatment with 0.75% EG and 1% AC, nearly all exhibited the deposition of calcium oxalate crystals in the kidneys [48]. Ethylene glycol undergoes oxidation to oxalic acid via non-specific dehydrogenase activity, leading to hyperoxaluria, a crucial factor in urolithiasis. Furthermore, EG undergoes metabolic processes leading to the formation of calcium oxalate monohydrate, resulting in renal mitochondrial toxicity closely resembling that observed in clinical calcium oxalate renal calculi [49]. In this study, male rats were specifically chosen to ensure the efficient formation of calcium oxalate stones. It has been documented that the male sex hormone testosterone has been observed to promote the formation of calcium oxalate crystals in adult rats treated with EG [50]. Furthermore, Research indicates that in the EG-induced animal model, the duration of stone deposition in female rats is comparatively shorter than in male rats [51]. Therefore, the present study predominantly employs male rats.

In this study, urolithiatic rats demonstrated a significant reduction in food intake, feed efficiency ratio, and body weight gain compared to the normal control group, indicating preliminary signs of renal toxicity, consistent with previous research findings [52]. However, administration of both the emulsion and nanogel emulsion significantly alleviated these detrimental reductions. This preventive effect on declining body weight can be attributed to their capability to counteract the toxic effects caused by ethylene glycol and ammonium chloride. EG’s final metabolite, oxalic acid, predominantly impact the kidneys, leading to acute poisoning symptoms [53]. The induction of renal stones in urolithiatic animals resulted in decreased food consumption, contributing to the decline in their body weight. Additionally, it is noteworthy that the urine of urolithiatic rats exhibited a significant presence of oxalate crystals, a phenomenon observed in various previous studies [54, 55].

Furthermore, the urolithiatic control group exhibited significant changes, marked by a notable decrease in urine volume and pH, coupled with an increase in the count of urinary calcium oxalate crystals. Additionally, this group demonstrated a substantial elevation in the urinary excretion of calcium, phosphate, and oxalate ions, along with a notable reduction in excreted magnesium levels compared to the normal control group. Following ethylene glycol administration, evident induction of hyperoxaluria and nephrotoxicity were observed. This was substantiated by histopathological examinations, which revealed oxalate crystal deposition within renal tubule lumens, accompanied by inflammatory responses and necrotic changes. This evidence was further supported by a significant elevation in immunohistochemical levels of inflammatory mediators such as TNF-α, as well as indicators of cytotoxicity and cell death induction, notably cleaved caspase-3. These findings are consistent with previous studies reporting tubular hypertrophy, tubulointerstitial damage, and extensive calcium oxalate crystal deposition in kidney tubules across various urolithiatic rat models [52].

The primary mechanism behind EG-induced stone formation is attributed to hyperoxaluria, leading to increased renal excretion and retention of oxalate [56]. This disrupts the equilibrium between lithogenesis promoters such as phosphate, oxalate, calcium, uric acid, and inhibitors like magnesium [57]. The heightened urinary phosphate excretion, in combination with oxalate stress, fosters an environment conducive to stone formation by primarily generating calcium phosphate crystals, thus promoting calcium oxalate deposition [58]. Improperly acidic or alkaline urine directly affects the solubility of various metabolites and salts in the body. Alkaline urine tends to reduce the solubility of calcium phosphate products, whereas an acidic pH in urine encourages the formation of stones containing calcium oxalate, uric acid, and cystine. Research has emphasized the pivotal role of urinary pH in kidney stone formation, suggesting that maintaining a urine pH around 6 on the pH scale significantly decreases the risk of stone development. Investigations on the risk of calcium oxalate crystallization across varying urine pH levels, involving individuals with and without a history of recurrent calcium oxalate stones, have shown that the highest risk of crystallization occurs within the pH range of 4.5 to 5.5 [57]. Consequently, the observed elevation in oxalate, calcium, and uric acid levels in this study triggered crystallization and subsequent precipitation of calcium oxalate within nephrons, causing damage to renal epithelial cells. These findings collectively highlight the intricate interplay of factors contributing to urolithiasis pathogenesis.

The administration of either the emulsion or nanogel emulsion had noticeable effects, including increased urine volume and pH, a reduction in urinary calcium oxalate crystals, decreased excretion of calcium, oxalate, and phosphate, and enhanced elimination of urinary magnesium. These effects might arise from the dual functionality of *C. proximus*, known for its diuretic and renal antispasmodic properties [10]. Moreover, the potent antioxidative activity found in parsley seeds, attributed to bioactive compounds like flavonoids and carotenoids [17], potentially enhances antioxidant defenses, raises kidney glutathione levels, and aids in kidney tissue restoration following nephrotoxicity [19]. Additionally, the anti-inflammatory properties of GA and its ability to protect nephrons might also contribute to these observed effects [21].

Regarding the clinical aspect, our urolithiatic animal models displayed notably increased serum levels of creatinine, BUN, urea, and uric acid compared to the normal control group. These findings strongly suggest severe impairment of kidney function, primarily due to reduced clearance of waste products from the bloodstream to the urine. This is attributed to both diminished glomerular filtration rate and damage to renal tubular cells. The obstruction caused by stones within the urinary system significantly reduced the glomerular filtration rate [54]. Concurrently, damage to proximal tubule cells hindered the efficient clearance of waste products, especially nitrogenous substances like blood creatinine, urea, and uric acid. Consequently, this led to an increased accumulation of these waste products within the bloodstream [59, 60], intensifying renal damage and further compromising kidney function.

Elevated urinary uric acid levels were also notably observed in our urolithiatic animals, associated with an increased risk of stone formation due to its role as a crystallization promoter [61]. This association is due to the ability of uric acid-binding proteins to interact with calcium oxalate, altering its crystallization kinetics, emphasizing its significant role in stone formation [62]. However, it’s noteworthy that administration of either the emulsion or nanogel emulsion, along with EG and AC, significantly reduced serum concentrations of creatinine, urea, uric acid, and BUN compared to the urolithiatic rat group. These changes are attributed to an improvement in the clearance of blood creatinine, urea, and uric acid into the urine following intervention.

Our findings align with previous studies conducted by Al-Yousofy et al. (2017) [63] and El-Nabtity et al. (2019) [64]. These investigations focused on a rat model of stone formation induced by administering 0.75% ethylene glycol and 2% ammonium chloride in drinking water over 10 days. The experiments revealed that treatment with either ethanolic or aqueous extracts of *C. proximus* or *P. crispum* exhibited notable nephroprotective and antiurolithiatic effects. The treated groups showed significantly lower levels of creatinine, blood urea nitrogen, and calcium compared to the stone-induction group. These extracts demonstrated antiurolithiatic properties through various mechanisms: 1- Reduced Urinary Calcium Excretion: Decreasing calcium excretion in urine lowers the risk of calcium oxalate crystallization. 2- Increased Urinary pH: Elevating urinary pH levels creates an unsuitable environment for calcium oxalate crystallization. 3- Diuretic Effect: Inducing diuresis increased urine volume, reducing urine supersaturation with stone-forming compounds. 4- Nephroprotective Activity: Additionally, these extracts exhibited nephroprotective properties, shielding the kidneys from adverse effects associated with stone formation.

Moreover, Gumaih et al. (2017) [65] evaluated the antiurolithiatic effect of parsley in rats with EG-induced urolithiasis, reported a significant reduction in serum urea, creatinine, uric acid, and electrolytes, along with a notable decrease in urinary calcium and proteins within the treated group. The authors attributed parsley’s nephroprotective effects to its antioxidant activity derived from its high content of flavonoids. These bioactive substances play a crucial role in preventing free radical damage induced by ethylene glycol. Furthermore, the therapeutic potential of GA in kidney diseases has been documented. Patients suffering from chronic kidney diseases who received GA demonstrated significant reductions in serum urea, creatinine, and levels compared to baseline and the control group [66].

The exposure of renal epithelial cells to elevated oxalate levels and the presence of calcium oxalate crystals often result in excessive reactive oxygen species (ROS) generation, leading to cellular injury and inflammation [67]. The administration of EG and AC heightened the levels of MDA, a marker of lipid peroxidation, while reducing reduced glutathione levels and CAT enzyme activity. However, the administration of either the emulsion or its nanoform showcased remarkable antioxidant activity, as evidenced by reduced MDA levels and an increase in antioxidant enzyme activities. These findings correspond with existing literature consistently reporting oxidative stress and lipid peroxidation in stone formation contexts [68, 69]. In a study by Ali et al. (2020) [66], GA exhibited a substantial impact on patients undergoing hemodialysis. Specifically, it significantly elevated total antioxidant capacity levels while notably reducing oxidative markers like MDA and C-reactive protein. These compelling findings robustly support the potent anti-inflammatory properties attributed to GA.

The GC-MS analysis of the ethanolic extracts of *P. crispum* seeds combined with *C. proximus* ethanolic extracts revealed a rich composition of phytochemicals and bioactive compounds. Major identified components included polyphenols, terpenoids, carotenes, porphyrin, milbemycin, fucoxanthin, and cyanocobalamin, recognized for their well-established antioxidant and anti-inflammatory properties. These ethanolic extracts exhibited a notable antiurolithiatic effect against calculi induced by EC and AC in this study. The observed efficacy in mitigating urolithiasis likely stems from the identified bioactive compounds within the extracts, highlighting their potential therapeutic relevance in addressing urolithiasis and promoting renal health. Previous research supports the inhibitory effects of terpenoids on the formation and size of calcium oxalate crystals [70]. Additionally, porphyrin has shown anti-ROS enzyme-mimicking capabilities, alleviating ROS-induced cell apoptosis and improving renal function in acute kidney injury mice [71]. Lutein, as reported by Hu et al. (2022) [72], displayed antioxidant properties, associated with a lower mortality risk in the chronic kidney disease population due to high-level carotene dietary intake. Fucoxanthin, highlighted by Wang et al. (2020) [73], emerged as a compound with significant antiurolithiatic properties, restoring antioxidant levels, regulating stone and renal markers, and preventing glomerular and tubular damage in lithiatic rats induced by Ethylene glycol. Milbemycins were found to be effective fungal growth inhibitors [74]. Furthermore, vitamin B12 exhibited antioxidant properties, protecting cells and DNA from reactive oxygen species-induced damage, inversely correlating with the risk of kidney stone development [75]. Additionally, Ke et al. (2023) [76], emphasized the association between a higher Oxidative Balance Score, indicative of an antioxidant-skewed balance, and a reduced risk of kidney stones, particularly among specific population subgroups.

Our study investigated the anti-urolithic effects of *C. proximus* and *P. crispum* seed ethanolic extract/GA emulsion, and nanogel emulsion, against EG and AC-induced urolithiasis in rats. The findings revealed that treatment with either the emulsion or nanogel emulsion significantly prevented urolithiasis-related abnormalities, including decreased urinary excreted magnesium and non-enzymic antioxidant glutathione, as well as catalase activity. Additionally, both treatments reduced oxalate crystal numbers, excretion of urolithiasis promoters, renal function parameters, and lipid peroxidation, while improving histopathological changes. Notably, the nanogel emulsion exhibited superior effects compared to the emulsion, as evidenced by decreased renal crystal deposition score and reduced expression of TNF-α and cleaved caspase-3. This observation suggests that converting the treatment into a nano-sized form could potentially enhance its bioavailability, effectiveness, and therapeutic action. Previous literature has emphasized the excellent biocompatibility of lipid-based nanoparticles [77]. The transformation of the emulsion into a nanogel emulsion improved the bioavailability of bioactive compounds, resulting in a more pronounced reduction in urolithiasis and nephrotoxicity.

## Conclusion

Our study has unveiled novel approaches for managing urolithiasis, demonstrating the potential of *C. proximus* and *P. crispum* seeds extracts/GA emulsion, as well as their nanogel form, as antiurolithiatic agents. These compounds exhibit multifaceted mechanisms, including reducing urinary calcium excretion, and elevating urinary pH levels, inducing diuresis, thereby preventing urine supersaturation and oxalate crystal formation. Both the emulsion and nanogel formulations show promise in improving kidney function, ameliorating urine and serum parameters, mitigating histopathological changes, and alleviating oxidative stress and inflammation associated with urolithiasis, with the nanogel emulsion demonstrating superior efficacy.

## Electronic supplementary material

Below is the link to the electronic supplementary material.

Supplementary Material 1

## Data Availability

The authors confirm that the data supporting the findings of this study are available within the article.
