# Supplementary material for: Cymbopogon proximus and Petroselinum crispum seed ethanolic extract/Gum Arabic nanogel emulsion: Preventing ethylene glycol and ammonium chloride-induced urolithiasis in rats
Source: Urolithiasis. 2024 Apr 2;52(1):52. doi: 10.1007/s00240-024-01559-2 (PMC10987356; doi:10.1007/s00240-024-01559-2)
Supplement: Supplementary file 1 — Supplementary Material 1 [file 240_2024_1559_MOESM1_ESM.pdf]

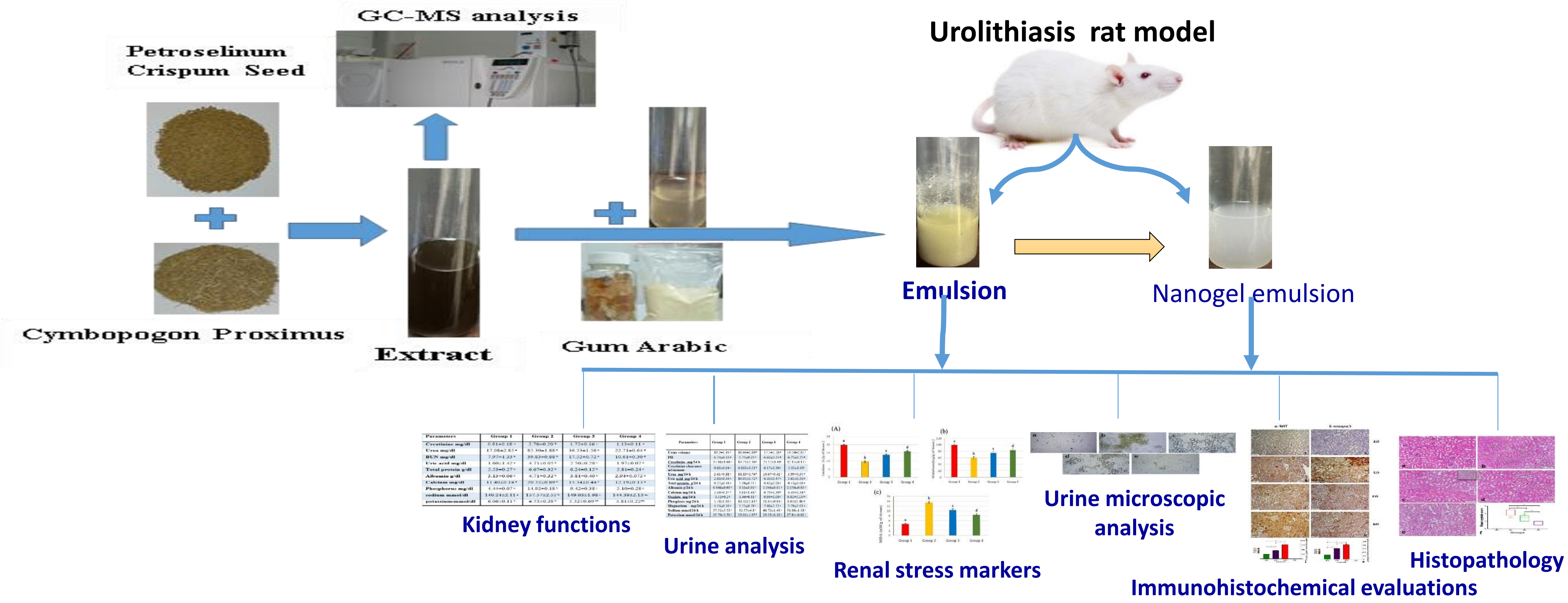
GC-MS analysis
Petroselinum Crispum Seed
Cymbopogon Proximus
Extract
Gum Arabic
Urolithiasis rat model
Emulsion
Nanogel emulsion
Kidney functions
Urine analysis
Renal stress markers
Urine microscopic analysis
Immunohistochemical evaluations
Histopathology
